# Glucose-Dependent Insulinotropic Polypeptide and Substance P Mediate Emetic Response Induction by Masked Trichothecene Deoxynivalenol-3-Glucoside through Ca^2+^ Signaling

**DOI:** 10.3390/toxins14060371

**Published:** 2022-05-27

**Authors:** Zihui Qin, Hua Zhang, Qinghua Wu, Ben Wei, Ran Wu, Xinyi Guo, Huiping Xiao, Wenda Wu

**Affiliations:** 1 MOE Joint International Research Laboratory of Animal Health and Food Safety, College of Veterinary Medicine, Nanjing Agricultural University, Nanjing 210095, China; 2019107104@njau.edu.cn (Z.Q.); 2020107103@stu.njau.edu.cn (B.W.); wuranvicky@163.com (R.W.); 2021107103@stu.njau.edu.cn (X.G.); huiping0729@163.com (H.X.); 2 School of Animal Husbandry and Veterinary Medicine, Jiangsu Vocational College of Agriculture and Forestry, Jurong 212400, China; zhanghuasd@163.com; 3 College of Life Science, Yangtze University, Jingzhou 434025, China; wqh212@yangtzeu.edu.cn; 4 Department of Chemistry, Faculty of Science, University of Hradec Kralove, 50003 Hradec Kralove, Czech Republic

**Keywords:** deoxynivalenol-3-glucoside, emesis, brain-gut peptide, calcium-sensing receptor, transient receptor potential channel

## Abstract

Deoxynivalenol (DON), the most naturally-occurring trichothecenes, may affect animal and human health by causing vomiting as a hallmark of food poisoning. Deoxynivalenol-3-glucoside (D3G) usually co-occurs with DON as its glucosylated form and is another emerging food safety issue in recent years. However, the toxicity of D3G is not fully understood compared to DON, especially in emetic potency. The goals of this research were to (1) compare emetic effects to D3G by oral and intraperitoneal (IP) routes and relate emetic effects to brain-gut peptides glucose-dependent insulinotropic polypeptide (GIP) and substance P (SP) in mink; (2) determine the roles of calcium-sensing receptor (CaSR) and transient receptor potential (TRP) channel in D3G’s emetic effect. Both oral and IP exposure to D3G elicited marked emetic events. This emetic response corresponded to an elevation of GIP and SP. Blocking the GIP receptor (GIPR) diminished emetic response induction by GIP and D3G. The neurokinin 1 receptor (NK-1R) inhibitor Emend^®^ restrained the induction of emesis by SP and D3G. Importantly, CaSR antagonist NPS-2143 or TRP channel antagonist ruthenium red dose-dependently inhibited both D3G-induced emesis and brain-gut peptides GIP and SP release; cotreatment with both antagonists additively suppressed both emetic and brain-gut peptide responses to D3G. To summarize, our findings demonstrate that activation of CaSR and TRP channels contributes to D3G-induced emesis by mediating brain-gut peptide exocytosis in mink.

## 1. Introduction

Deoxynivalenol (DON, also known as vomitoxin), a type B trichothecene mycotoxin, is a secondary metabolite of varieties of *Fusarium* species, found mostly in cereals and cereal-based products [1,2,3]. Its presence can cause many detrimental impacts on plants, animals and humans. The occurrence of DON in food, feed and agricultural products and exposure risk assessment in humans have been reported globally [4,5]. Moreover, the profiles of human DON biomarkers have been systematically assessed in different regions, ages, sexes, and dietary habits all over the world [6]. In 2021, Biomin examined 16,164 finished feed and raw commodity samples from 74 countries across the world. Contamination rates by continent were as follows: 68% in North America, 68% in Central America, 46% in South America, 52% in Europe, 58% in the Middle East, and 81% in Sub-Saharan Africa [7]. Furthermore, risk exposure is extreme in South Asia, China and Taiwan. Corn stays heavily affected with a very high abundance of DON (80%). Obviously, DON is one of the most common toxins. In humans and animals, DON elicits acute toxicities, including anorexia, emesis, diarrhea, gastroenteritis, endotoxemia, and even shock-like death [8,9,10,11].

Host plants excrete a glucosylation enzyme onto the plant surface that changes mycotoxins derived from fungal secondary metabolites to glucosylated products. DON-3-glucoside (D3G) is synthesized by grain uridine diphosphate-glucosyltransferase and is found worldwide [12]. Free DON and its acetylated forms are naturally occurring fungal secondary metabolites, whereas D3G is a conjugate of DON formed in plants [13]. Furthermore, in plants, phase II metabolism is characterized by detoxification because of conjugation reactions that take place through the combination of hydrophilic biomolecules with DON or phase I metabolic products, catalyzed by glycosyl-transferases and other enzymes [14]. Additionally, it was reported that D3G cannot bind to ribosomes; therefore, it neither activates mitogen-activated protein kinase (MAPK) nor induces inflammation owing to the steric hindrance of the glucoside moiety [15]. Compared with DON, D3G shows lower toxicity in humans; however, it is biotransformed into DON when it is ingested into the human digestive tract [16,17]. The incubation of D3G with pig intestinal microbiota can realize the hydrolysis process mentioned above [18]. In summary, D3G can be effectively hydrolyzed in the intestinal tract resulting in an increase of its aglycon and, hence, posing a potential risk to exposed individuals. Importantly, the risk to animal and human health related to D3G has been assessed in food and feed, indicating a potential health concern [2]. Besides this, recent advances in human D3G biomarkers are also worth a mention [6].

Despite increasing concern about the toxic effects of D3G, the toxicological data on this mycotoxin are still scarce. In addition to these discoveries mentioned above, the role of D3G in inducing anorexia and the underlying mechanism by which the brain-gut peptides peptide YY (PYY) and cholecystokinin (CCK) are triggered were discovered in our prior research [19]. D3G, simultaneously, also showed emetic potential after oral administration in mink. However, the impacts of D3G on emesis and brain-gut peptide secretion remain not fully defined. We have also found that DON dose-dependently evoked emesis in mink following both orally and IP dosing [20,21]. Subsequently, we demonstrated that DON elicited brain-gut peptide-driven anorexia and emesis in a murine anorexia model and mink emesis model by activating the Calcium-sensing receptor (CaSR) and a transient receptor potential (TRP) channel named TRP ankyrin 1 (TRPA1), respectively [22,23]. CaSR and TRP channels situated on enteroendocrine cells (EECs) are commonly used as chemosensors for gastrointestinal contents [24,25]. As reported, in a murine EEC model, exposure to DON can promote the secretion of brain-gut peptides glucagon-like peptide-1 (GLP-1) and CCK by activating CaSR and TRPA1-mediated Ca^2+^ signaling [26]. Nevertheless, the functions of CaSR and TRP channel in emetic potency of D3G are still unclear. Further research is needed as a matter of priority. Specifically, the aim of this study was to link the emetic capacity of D3G to the generation of brain-gut peptides including glucose-dependent insulinotropic polypeptide (GIP) and substance P (SP) and to clarify that D3G induces an emetic response by stimulating CaSR and TRP channels in mink.

## 2. Results

### 2.1. Emetic Potencies of D3G following Oral and IP Dosing

**Figure 1:**
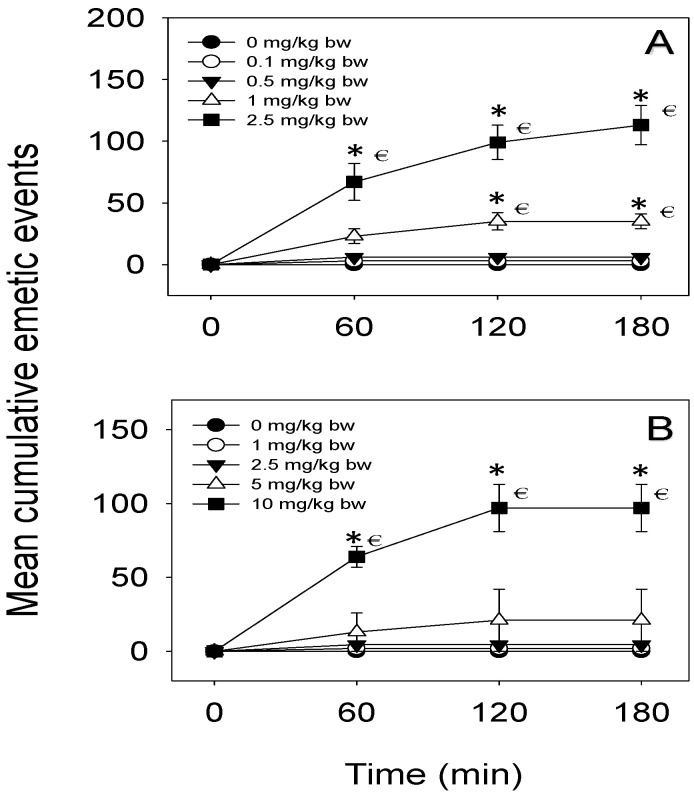
Mean cumulative emetic events following (**A**) oral and (**B**) IP exposure to D3G in mink. Data represent mean ± SEM (*n* = 5/group). Two-way ANOVA using the Holm–Sidak method was used to assess significant differences in mean cumulative emetic events, as compared with the control. Symbols: * indicates a statistically significant difference in cumulative emetic episodes compared with the control (*p* < 0.05) and € indicates a statistically significant difference relative to the 0-min time point within a given dose (*p* < 0.05).

Emetic potencies of oral and IP dosing with D3G were exhibited (Table 1). After oral treatment, 0.1 and 0.5 mg/kg bw D3G displayed no effect (Figure 1A); 1 mg/kg bw is the lowest emetic dose of D3G, which induces a response in 40% of the mink. When mink were orally treated with 2.5 mg/kg bw D3G, all the mink vomited. Most of the vomiting occurred within 120 min and lasted up to 180 min. Upon IP injection, 1 and 2.5 mg/kg bw D3G displayed no effect (Figure 1B); 5 mg/kg bw is the lowest emetic dose of D3G, which caused a response in 20% of the mink. When the dose of D3G increased to 10 mg/kg bw, all the mink vomited. Most of the emetic episodes happened during 60–120 min and lasted for up to 180 min.

### 2.2. D3G-Induced Emetic Effect Corresponds to Elevation of GIP and SP

**Figure 2:**
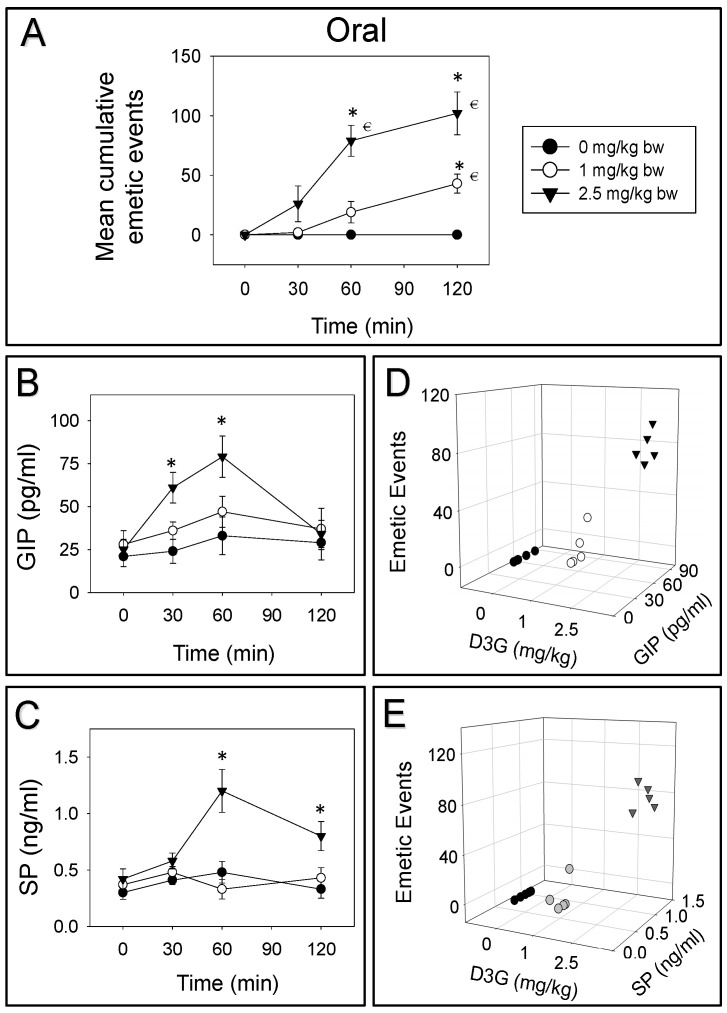
Emetic response corresponds to an elevation of GIP and SP by oral exposure to D3G. (**A**) Mean cumulative emetic events in mink following oral exposure to D3G. Kinetics of D3G-induced (**B**) GIP and (**C**) SP elevation in plasma. Relationship between emetic events and (**D**) GIP, (**E**) SP levels at 60 min. Data are mean ± SEM (*n* = 5/group). Two-way ANOVA using the Holm–Sidak test was used to analyze significant differences in mean cumulative emetic events and kinetics of GIP and SP in mink. Symbols: * indicates a statistically significant difference in mean cumulative emetic events and GIP or SP concentration relative to the control at a specific time point (*p* < 0.05). € indicates a statistically significant difference in mean cumulative emetic events relative to the 0 min time point (*p* < 0.05). The Spearman rank-order correlation coefficient was used for the correlation between emetic events and hormone levels (*p* < 0.05).

Mink were orally gavaged and IP injected with D3G, and then GIP, SP were tested over 180 min. Upon oral treatment of 1 or 2.5 mg/kg bw D3G, 5%, 40% and 55% or 24%, 54% and 22% vomiting occurred during 0–30, 30–60 and 60–120 min periods, respectively (Figure 2A). Following oral treatment of 2.5 mg/kg bw D3G, plasma GIP was elevated at 30 and 60 min (Figure 2B). After 120 min, GIP returned to basal level. Whereas, 1 mg/kg bw D3G displayed no effect on GIP. Plasma SP was raised at both 60 and 120 min by orally dosing with 2.5 mg/kg bw D3G, while 1 mg/kg bw D3G displayed no effect on SP (Figure 2C). Dose-dependent elevation of GIP and SP corresponded to emetic episodes triggered by oral exposure to D3G at 60 min (Figure 2D,E).

**Figure 3:**
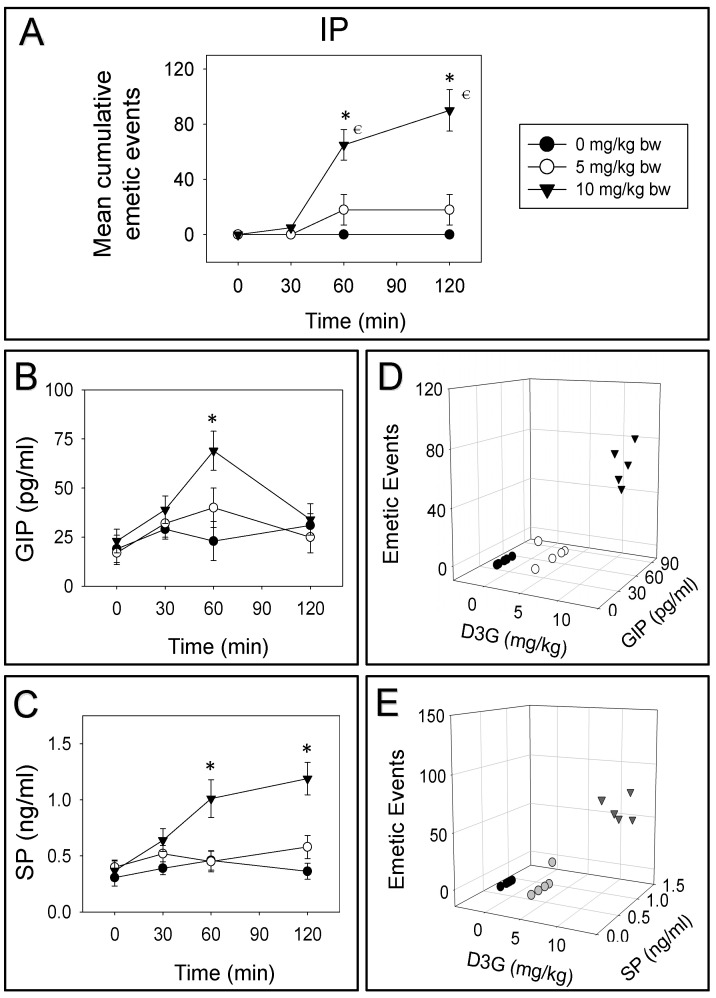
D3G-induced emetic response (**A**) corresponds to an elevation of GIP (**B**,**D**) and SP (**C**,**E**) following IP exposure. Experiment carried out and data assessed as depicted in Figure 2 legend. Symbols: * indicates a statistically significant difference in mean cumulative emetic events and GIP or SP concentration relative to the control at a specific time point (*p* < 0.05). € indicates a statistically significant difference in mean cumulative emetic events relative to the 0 min time point (*p* < 0.05). The Spearman rank-order correlation coefficient was used for the correlation between emetic events and hormone levels (*p* < 0.05).

IP dosing with 5 or 10 mg/kg bw D3G evoked 0%, 100% and 0% or 6%, 65% and 29% vomiting during 0–30, 30–60 and 60–120 min periods, respectively (Figure 3A). Upon IP exposure to 10 mg/kg bw D3G, plasma GIP was increased only at 60 min (Figure 3B). Whereas, 5 mg/kg bw D3G displayed no effect on GIP. Plasma SP was raised at both 60 and 120 min by IP dosing with 10 mg/kg bw D3G, while 5 mg/kg bw D3G displayed no effect on SP (Figure 3C). Dose-dependent elevation of GIP and SP in plasma corresponded to emetic episodes stimulated by IP exposure to D3G at 60 min (Figure 3D,E).

### 2.3. Effects of Brain-Gut Peptide Receptor Inhibitor on D3G-Induced Emesis

**Figure 4:**
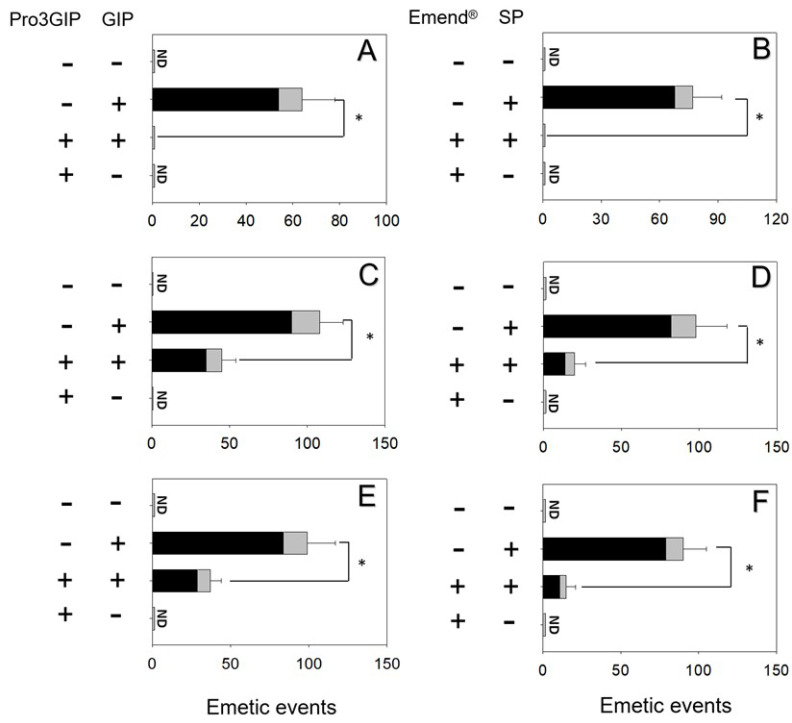
GIPR inhibitor Pro3GIP and NK-1R inhibitor Emend^®^ diminished emetic episodes induced by (**A**,**B**) GIP and SP, (**C**,**D**) D3G after oral treatment, (**E**,**F**) D3G after IP treatment, respectively. Emetic episodes contain retching (black) and vomiting (gray) episodes. ND = not detected. Data represent mean ± SEM (*n* = 5/group). A one-way ANOVA using Holm-Sidak was used to assess significant differences between treatments and the respective controls. Symbols: * indicates statistically significant differences in emetic episodes (*p* < 0.05).

The effects of blocking the GIPR in GIP- and D3G-induced emesis were evaluated. Mink dosed with GIP displayed 64 ± 14 emetic episodes (Figure 4A). Pretreatment of GIPR inhibitor Pro3GIP completely blocked GLP-1-induced emetic episodes. Given that Pro3GIP alone did not lead to vomiting, suggesting a lack of functional antagonism. Mink orally and IP dosed with D3G had 108 ± 15 and 99 ± 18 emetic episodes, respectively (Figure 4C,E). Receiving Pro3GIP prior to oral and IP exposure to D3G in mink diminished 58% and 62% emetic episodes, respectively.

The effects of blocking the NK-1R in SP- and D3G-induced vomiting were evaluated. Mink dosing with SP elicited 76 ± 16 emetic episodes (Figure 4B). Pretreatment of NK-1R antagonist Emend^®^ completely blocked emetic episodes induced by SP. Given Emend^®^ alone did not lead to vomit, suggesting a lack of functional antagonism. Animals orally and IP treated with D3G had 98 ± 20 and 90 ± 15 emetic episodes, respectively (Figure 4D,F). Receiving Emend^®^ prior to oral and IP exposure to D3G in mink diminished 80% and 84% emetic episodes, respectively.

### 2.4. Roles of CaSR and TRP Channel on D3G-Induced Emesis and Brain-Gut Peptides

**Figure 5:**
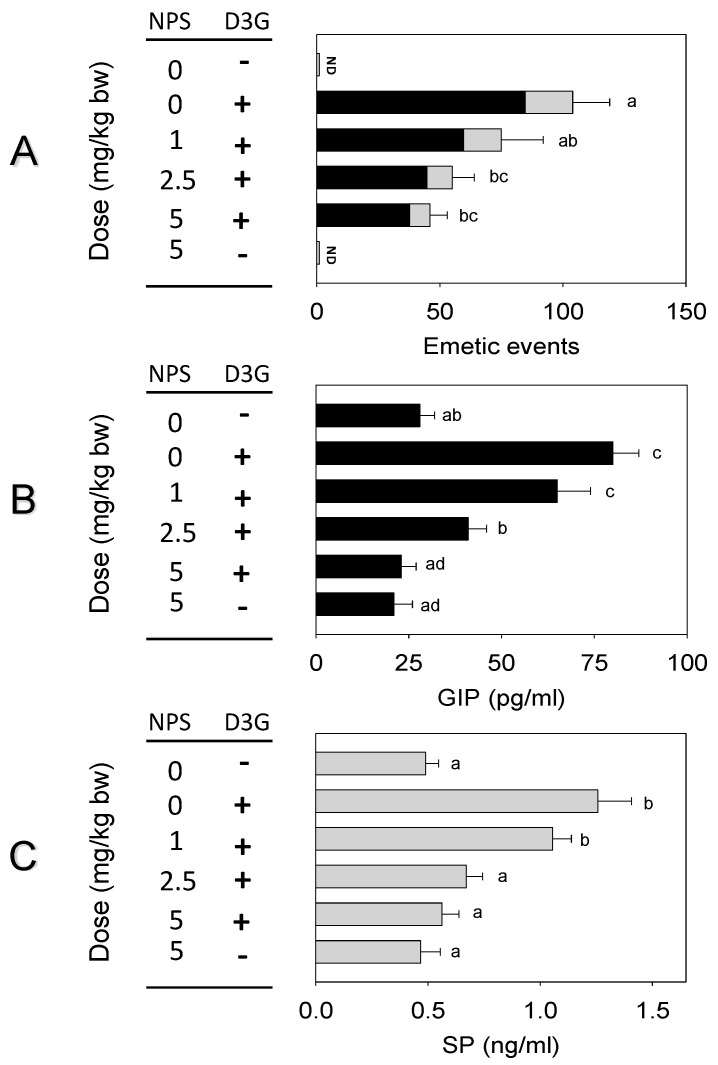
D3G-induced emetic episodes (**A**), GIP (**B**) and SP (**C**) were dose-dependently attenuated by CaSR antagonist NPS. Emetic episodes contain retching (black) and vomiting (gray) episodes. ND = not detected. Data represent mean ± SEM (*n* = 5/group). A one-way ANOVA using Holm-Sidak was used to analyze significant differences between multiple groups. Bars without the same letter are significantly different (*p* < 0.05).

**Figure 6:**
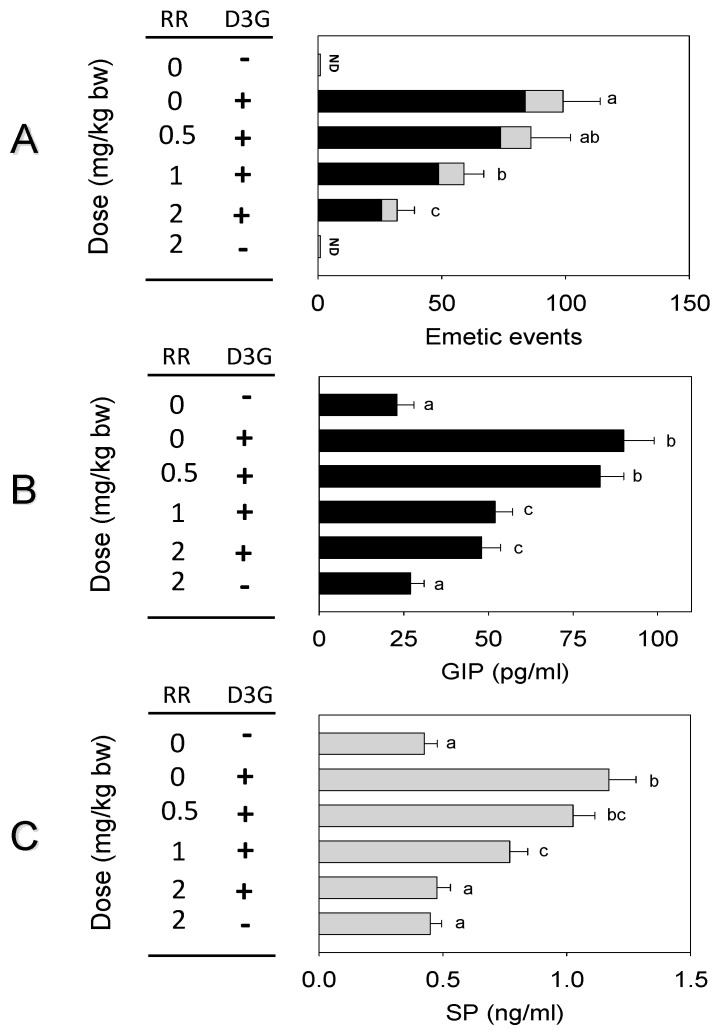
D3G-induced emetic episodes (**A**), GIP (**B**) and SP (**C**) were dose-dependently attenuated by TRP channel antagonist RR. The experiment was carried out and the data was assessed as depicted in Figure 5 legend.

The impact of CaSR antagonist NPS on D3G-induced emesis was assessed in the mink. Oral exposure to D3G at 2.5 mg/kg bw caused 104 ± 15 emetic episodes (Figure 5A). Pretreatment with NPS reduced 27, 47, and 56% of emetic episodes at 1, 2.5 and 5 mg/kg bw, respectively. Plasma GIP and SP concentrations raised significantly 1 h after oral exposure to D3G. Pre-dosed with 1, 2.5 and 5 mg/kg bw NPS reduced 19, 49, and 71% of GIP or 16, 46, and 55% of SP, respectively (Figure 5B,C). When mink were pre-dosed with TRP channel antagonist RR, emetic episodes were decreased by 13, 41 and 67% at 0.5, 1 and 2 mg/kg bw, respectively (Figure 6A). Pretreatment with 0.5, 1 and 2 mg/kg bw RR, plasma GIP or SP declined by 8, 43 and 45% or 12, 34 and 60%, respectively (Figure 6B,C).

**Figure 7:**
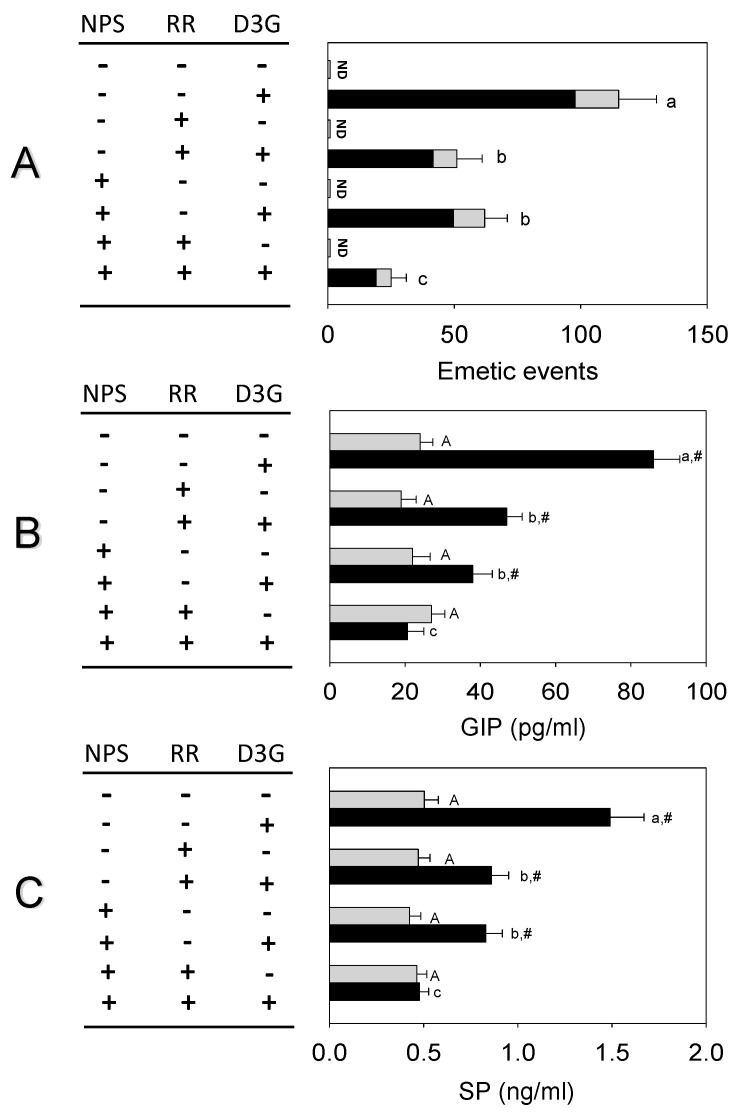
CaSR antagonist NPS and TRP channel antagonist RR additively inhibit D3G-induced emetic episodes (**A**), GIP (**B**) and SP (**C**) in mink. Emetic episodes contain retching (black) and vomiting (gray) episodes. ND = not detected. Data represent mean ± SEM (*n* = 5/group). A one-way ANOVA using Holm-Sidak was used to analyze significant differences between multiple groups. Bars without the same letter are significantly different (*p* < 0.05). Statistical comparisons between two groups were analyzed using Student’s *t*-test. The # sign indicates a significant difference between vehicle-treated and corresponding D3G-treated groups (*p* < 0.05).

The combined effects of NPS and RR on D3G-induced emetic response and brain-gut peptides release were shown in Figure 7. Upon pre-dosed with 2.5 mg/kg bw NPS or 1 mg/kg bw RR, D3G-induced emetic episodes were attenuated by 46 and 55%, respectively (Figure 7A). When mink were treated with both antagonists, emetic episodes were attenuated by 76%. Plasma GIP and SP concentrations increased significantly 1 h after oral exposure to D3G. Pretreatment with 2.5 mg/kg bw NPS or 1 mg/kg bw RR and D3G-induced GIP were attenuated by 57 and 45%, respectively (Figure 7B). When mink were treated with both antagonists, plasma GIP was attenuated by 77%. Pretreatment with 2.5 mg/kg bw NPS alone, D3G-induced SP was attenuated by 43% (Figure 7C). Mink treated with 1 mg/kg bw RR alone had a significant decrease in plasma SP (44%). It showed a significant decrease in plasma SP (68%) when mink were treated with both antagonists.

## 3. Discussion

Vigorous and continuous vomiting can disrupt the electrolyte and nutrient absorption balance and seriously affect health [27]. There are brain-gut peptides, vagal afferent neurons, and the central pattern generator (CPG, also known as the “emesis center”) involved in vomiting regulation through central and peripheral pathways [28,29]. In the peripheral pathway, vomiting agents trigger intestinal EEC to release brain-gut peptides, which bind to their specific receptors on the vagus nerve. In the central route, vomiting agents stimulate brain EEC cells to secrete brain-gut peptides, which bind to specific receptors located in the posterior region of the brain [30]. Signals are then sent to the nucleus tractus solitarius (NTS), activating CPGs and ultimately causing vomiting [31]. Vomiting is a sign of food poisoning caused by trichothecenes including D3G, but its pathological mechanisms remain unclear. Previous research in mink has shown that both D3G and its patent toxin DON could elicit robust emetic episodes [19,21]. This is the first research to relate the emetic potency of D3G to brain-gut peptides (GIP and SP) and elucidate the roles of CaSR and TRP channels in vomiting. The results revealed three novel findings (1) D3G-induced vomiting corresponded to the secretion of GIP and SP; (2) GIP and SP mediated vomiting via GIPR and NK-1R dependent mechanisms; (3) D3G triggered emesis in mink by activating CaSR and TRP channel-mediated Ca^2+^ signaling.

This is the first study to demonstrate that D3G has the ability to cause elevation of GIP and SP, which mediate emetic response in mink. This protein belongs to the incretin hormone family, GIP is composed of 42 amino acids and secreted by enteroendocrine K cells within the jejunum and duodenum [32]. Produced by enterochromaffin (EC) cells in the intestine and brain, SP is an 11 amino acid brain-gut peptide and belongs to the tachykinin family [33]. Many studies have reported that GIP and SP have the ability to elicit vomiting in humans and animals [34,35,36]. Based on currently available data, the estimates of acute dietary exposure to the sum of DON, 3-Ac-DON, 15-Ac-DON and D3G were below the acute reference dose of 0.008 mg/kg bw per eating occasion for humans and considered not a serious health problem [2]. A NOAEL has been estimated for vomiting in humans of 0.026 mg DON/kg bw per a single eating occasion. Moreover, some observations were conducted in prior research for pigs, which show NOAEL and LOAEL of DON as 0.075 and 0.1 mg/kg bw or 0.025, and 0.05 mg/kg bw for orally or IP dosing, respectively [37]. In mink, the orally or IP dosing for NOAEL and LOAEL of DON are 0.01 and 0.05 mg/kg bw or 0.05, and 0.1 mg/kg bw, respectively [21]. For vomiting, NOAELs of 0.026, 0.075 and 0.01 mg/kg bw for orally dosing were identified for DON in humans, pigs and mink, respectively. Furthermore, the emetic potencies of DON in different subjects may follow rank orders of mink > humans > pigs for oral exposure. Interestingly, DON was reported to have the potency to stimulate the secretion of GIP in rodents [38]. As DON’s congener, another trichothecene T-2 toxin also could trigger the secretion of GIP and SP in rodents [39,40]. Moreover, DON and T-2 toxin-evoked anorectic responses were involved in GIPR and NK-1R dependent mechanisms [41,42,43]. Other research also showed that GIP and SP could induce pica and delayed gastric emptying by activating GIPR and NK-1R in rodents [44,45,46]. Because the rodents lack CPG, anorectic response, pica and delayed gastric emptying in rodents are considered substitutes for vomiting, suggesting that GIP and SP might bind to GIPR and NK-1R to stimulate vomiting in humans and animals. In this study, D3G was demonstrated to trigger emetic episodes via GIPR and NK-1R dependent mechanisms, which is consistent with our previous findings in DON and T-2 toxins.

In this study, the orally or IP dosing for NOAEL and LOAEL of D3G are 0.5 and 1 mg/kg bw or 2.5 and 5 mg/kg bw, respectively. This suggested that orally dosing to D3G evoked stronger emetic effects than IP. The greater effect of D3G administered orally compared to IP might be due to the fact that the toxin can be effectively hydrolyzed in the intestine resulting in an increase of its aglycon, and DON has the potential for higher toxicity. Besides, this observation is consistent with our prior research in mink for DON, which shows its NOAEL and LOAEL as 0.01 and 0.05 mg/kg bw or 0.05, and 0.1 mg/kg bw for orally or IP dosing, respectively [21]. Because the orally or IP dosing for NOAELs and LOAELs of D3G and DON were identified, it shows that D3G has, in comparison, weaker emetic potency than DON. Moreover, whether latency or duration in vomiting, D3G presented longer hours. It seems to reveal that D3G can form DON by hydrolysis in the intestine causing both mycotoxins exposure to occur simultaneously after D3G was ingested in mink. This reaction process extends the time of D3G’s emetic effect. However, some research on pigs showed the opposite result via different routes, which indicated that orally dosing was less effective in stimulating vomiting [37,47]. This might be related to the animal species’ differences in the absorption, metabolism, distribution and bioavailability of D3G in oral or IP dosing. Moreover, it is reasonable that D3G binds to one or more unknown chemoreceptors with different affinities located in the gastrointestinal tract. The differences in the distribution of these receptors throughout the gastrointestinal or systemic tract and the differences in receptor-ligand affinity may be the reason for the observed differences in the efficacy of vomiting after different exposure routes to D3G. Other possible reasons included the composition and abundance of the microbiota in the gut of different animal species owing to mycotoxin’ capacity of reshaping gut microbial structure [48]. Interestingly, orally dosing to D3G elicited stronger secretion of brain-gut peptides than IP, which was also suggestive of more absorption of D3G by the oral route.

**Figure 8:**
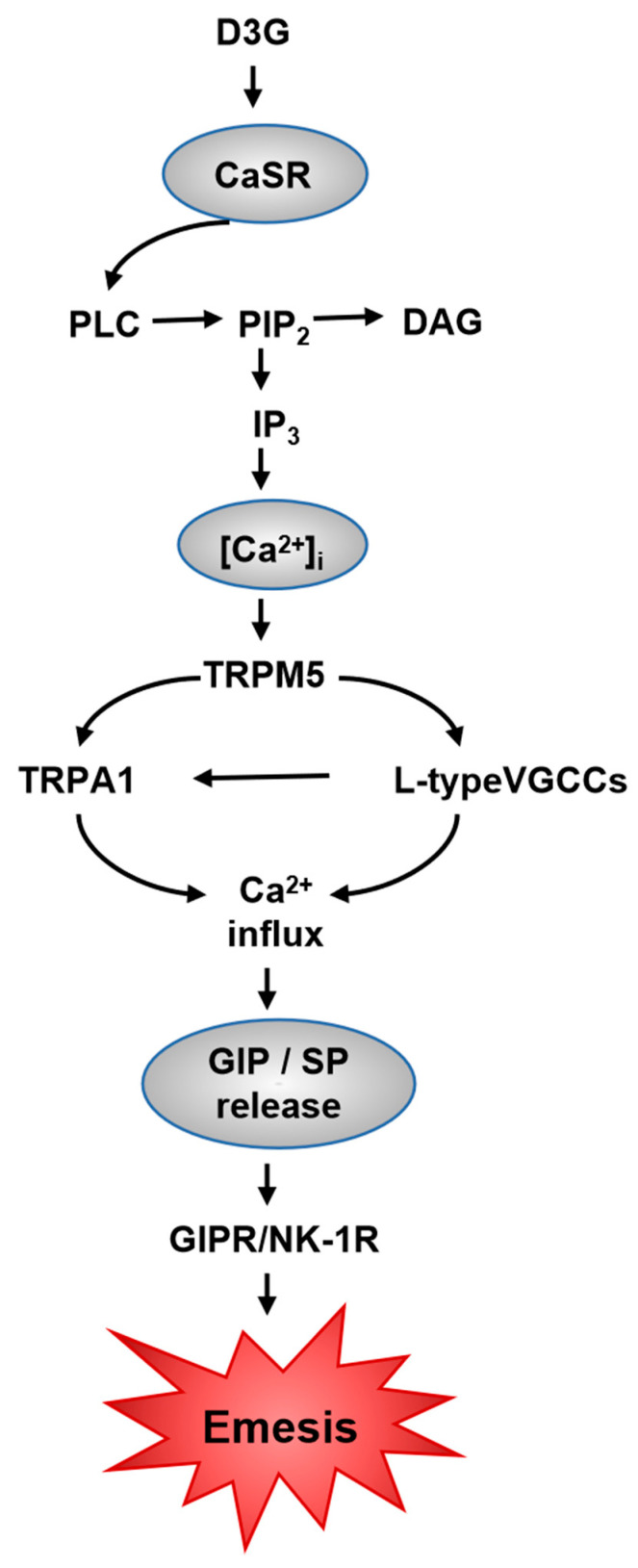
Putative role of CaSR and TRP channel on enteroendocrine cell (EEC) in D3G-induced emetic response.

In 2021, the discoverers of TRP channels were given the Nobel Prize in Physiology or Medicine, which indicates that the function of these receptors and channels is a potentially rewarding area for further study. TRP channels are multifunctional signaling molecules with many roles in sensory perception and cellular physiology [49]. Beyond that, in view of sequence homology, TRP channel superfamily contains seven subfamilies: TRPA, TRPC, TRPM, TRPN, TRPP, TRPML and TRPV [50]. Most TRPs are polymodal channels, so-called coincidence detectors that are activated by both chemical and physical (temperature, pressure, tension and voltage) stimuli [51]. CaSR, the C subfamily of the G protein-coupled receptor (GPCR) family, can be activated in response to extracellular Ca^2+^ [52]. The receptor is a primary role to maintain calcium (Ca^2+^) homeostasis in cellular and systemic physiological processes [53]. This paper demonstrates for the first time that the activation of CaSR and TRP channels is pivotal to D3G-triggered brain-gut peptide secretion which, in addition, can lead to vomiting. The putative pathway has been presented as shown in Figure 8. Moreover, in our previous studies, the activation of CaSR and TRPA1 was found to be the vital point for DON-induced brain-gut peptide exocytosis [22,23]. The specific pathway proposed is as follows: (1) CaSR irritation and phospholipase C-mediated activation of the IP3 receptor; (2) mobilization of intracellular Ca^2+^ reserves, TRPM5 activation and L-type voltage-sensitive Ca^2+^ channel-promoted extracellular Ca^2+^ entry; (3) TRPA1 activation and enhancement of extracellular Ca^2+^ entry; (4) exocytosis of brain-gut peptides driven by intracellular Ca^2+^. On the basis of the data provided here, we hypothesized that CaSR and TRP channels are also key calcium receptors and channels involved in mediating the toxicity of D3G, which is similar to its parent toxin DON.

## 4. Conclusions

To summarize, the data presented herein show that brain-gut peptides GIP and SP were robustly increased by orally and IP dosing with D3G. Emetic effects induced by this toxin were consistent with the elevation of GIP and SP via activation of GIPR and NK-1R. D3G induces emesis as well as brain-gut peptides elevation by activating both CaSR and TRP channels. Future investigations should focus on how DON other congeners act on primary EEC/EC to elicit GIP and SP exocytosis as well as the role of other TRP channel linkages to the molecular mechanisms of vomiting. From a public health perspective, research, such as this will improve our understanding of D3G and formulate strategies to prevent food poisoning from this toxin.

## 5. Materials and Methods

### 5.1. Animal and Reagent

1–2 years old Mink (1–1.5 kg, female) was obtained from Weifang City, Shandong Pr. in China (Far East Breeding Co., Ltd.) and housed in individual cages with 12 h light/dark cycle, 20–24 °C and 30–70% humidity. Guidelines for the animal study were established by the Institutional Animal Care and Use Committee at Nanjing Agricultural University (Certification No: SYXK (Su) 2011-0036). D3G was purified from wheat as previously described [54] and the purity (>96%) was measured by HPLC-tandem mass spectrometry (HPLC-MS/MS; Christian Doppler Laboratory, Vienna, Austria). GIP, SP and receptor inhibitors Pro3GIP, Emend^®^ were obtained and prepared according to prior studies [40,41]. NPS-2143 (NPS) and ruthenium red (RR) were purchased and prepared according to previous studies [22,23].

### 5.2. Experimental Design

Study 1: Emetic potencies of D3G following oral and IP dosing.

Mink (*n* = 5/group) were fasted for 24 h, and then allow to eat feed for 30 min before the experiment. Then, mink were orally gavaged 0, 0.1, 0.5, 1, and 2.5 mg/kg bw D3G using a sterile stainless steel gavage tube (16-G, 5-cm) or IP injected 0, 1, 2.5, 5, and 10 mg/kg bw D3G using a sterile needle (20-G, 2.54-cm), respectively. After that, emesis in mink was monitored over the subsequent 6 h. Each individual retch or vomit was counted as described previously [55].

Study 2: Effect of D3G on brain-gut peptides.

After fasting and re-feeding as previously mentioned, mink (*n* = 5/group) were orally dosed with D3G at 0, 1 and 2.5 mg/kg bw or IP injected by 0, 5 and 10 mg/kg bw in 100 µL PBS, respectively. Following intramuscular administration, the animals were anesthetized with 10 mg/kg bw ketamine at 0-, 60-, 120- and 180-min intervals. Blood was collected via cardiac puncture using EDTA vacuum and centrifuged 10 min (1000× *g*, 4 °C) to plasma for GIP and SP (Phoenix Pharmaceuticals; Burlingame, CA, USA) ELISA examination.

Study 3: Effects of brain-gut peptide receptor inhibitor on D3G-induced emesis.

To estimate that GIP receptor (GIPR) inhibitor Pro3GIP could attenuate D3G-induced vomiting, mink was firstly gavaged 0.25 mg/kg bw Pro3GIP. To determine neurokinin 1 receptor (NK-1R) inhibitor Emend^®^ could abate D3G-evoked emetic episodes, mink were first given 1 mg/kg bw Emend^®^ upon subcutaneous injection. After that, mink were fed for 30 min and provided 2.5 or 10 mg/kg bw D3G upon oral or IP dosing, respectively. GIP and SP were also IP injected into mink at 0.2 and 0.5 mg/kg bw as a positive control, respectively. Emetic episodes including retch and vomit were recorded over the subsequent 180 min.

Study 4: Roles of CaSR and TRP channel on D3G-induced emesis and brain-gut peptides.

To assess the effect of NPS or RR on D3G-induced emetic response, fasted mink (*n* = 5) were first orally gavaged with NPS (1, 2.5 and 5 mg/kg bw) or RR (0.5, 1 and 2 mg/kg bw) in 1 mL vehicle, respectively, and provided 50 g feed immediately. To evaluate the combined effects of NPS and RR on D3G-induced emetic responses, fasted mink (*n* = 5) were first gavaged with 2.5 mg/kg bw NPS or 1 mg/kg bw RR or both in 1 mL vehicle, respectively, and then provided 50 g of food. After 30 min, mink were gavaged with 2.5 mg/kg bw D3G and monitored for emesis over the next 3 h.

To learn the dose-response impact of NPS and RR on D3G-induced brain-gut peptides release, fasted mink (*n* = 5) were orally gavaged with NPS (1, 2.5 and 5 mg/kg bw) or RR (0.5, 1 and 2 mg/kg bw) in 1 mL vehicle, respectively. To evaluate the combined effects of NPS and RR on D3G-induced brain-gut peptides release, fasted mink (*n* = 5) were first orally gavaged with 2.5 mg/kg bw NPS-2143 and/or 1 mg/kg bw RR in 1 mL vehicle or vehicle alone, respectively. After 30 min, mink were gavaged with 2.5 mg/kg bw D3G. At experiment termination 2 h later, mink were anesthetized with 10 mg/kg bw ketamine. Blood was collected via cardiac puncture using EDTA vacuum and centrifuged 10 min (1000× *g*, 4 °C) to plasma for GIP and SP (Phoenix Pharmaceuticals; Burlingame, CA, USA) ELISA examination.

### 5.3. Statistics

Data were calculated by SigmaPlot (Jandel Scientific; San Rafael, CA, USA) except emetic dose (ED), which was calculated through SAS using the Proc Probit method. Specific statistical methods were carried out as described in figure legends and differences were significantly regarded at *p* < 0.05.

## Figures and Tables

**Table 1:** Comparison of emetogenic potentials upon oral and IP exposure to D3G.

Exposure Route	Dose (mg/kg bw)	Incidence (Responding/Tested)	Latency (min) ^A,B^	Duration (min) ^A,B^	Emetic Episodes ^C^		
					Retch	Vomit	Total
Oral	0	0/5	-	-	0 ± 0	0 ± 0	0 ± 0
	0.1	0/5	-	-	0 ± 0	0 ± 0	0 ± 0
	0.5	0/5	-	-	0 ± 0	0 ± 0	0 ± 0
	1	2/5	31 ± 3 ^a^	33 ± 2 ^a^	27 ± 4	8 ± 2	35 ± 6
	2.5 *	5/5	25 ± 4 ^a^	101 ± 9 ^b^	92 ± 10	21 ± 6	113 ± 16
IP	0	0/5	-	-	0 ± 0	0 ± 0	0 ± 0
	1	0/5	-	-	0 ± 0	0 ± 0	0 ± 0
	2.5	0/5	-	-	0 ± 0	0 ± 0	0 ± 0
	5	1/5	37 ± 0 ^a^	9 ± 0 ^a^	16 ± 16	5 ± 5	21 ± 21
	10 *	5/5	32 ± 5 ^a^	82 ± 6 ^b^	83 ± 10	14 ± 6	97 ± 16

^A^ Average of positive responders only. ^B^ If animals did not elicit emetic episodes, latency and duration are displayed as “-”. ^C^ Average of both non-responders and responders. Data are mean ± SEM. * indicate significant differences at *p* < 0.05 for incidence, retch, vomit and total emetic episodes compared with the control. Different letters within a column indicate significant differences at *p* < 0.05.

## Data Availability

The data presented in this study are available in this article.
